# Berberine prevents postoperative recurrence of colorectal cancer: a protocol for a multicenter, double-blind, randomized controlled trial

**DOI:** 10.3389/fmed.2026.1908350

**Published:** 2026-08-13

**Authors:** Yulin Li, Lingyu Zhang, Han Tong, Zhixiang Xu, Yanghong Wei, Ziyang Lin, Da Wang, Xinjie Zhang, Linhao Tong, Jiakuan Bao, Hui Wu, Fangyue Xu, Changhong Wu, Haitao Chen

**Affiliations:** 1 The Second Clinical Medical College of Zhejiang Chinese Medical University, Hangzhou, Zhejiang, China; 2 The Third Clinical Medical College of Zhejiang Chinese Medical University, Hangzhou, Zhejiang, China; 3 Department of Colorectal Surgery and Oncology, Key Laboratory of Cancer Prevention and Intervention, Ministry of Education, The Second Affiliated Hospital, Zhejiang University School of Medicine, Hangzhou, Zhejiang, China; 4 Department of Oncology, Lishui Central Hospital (The Fifth Affiliated Hospital of Wenzhou Medical University, Lishui Hospital of Zhejiang University), Lishui, Zhejiang, China; 5 Department of Colorectal Surgery, The Second Affiliated Hospital of Zhejiang University School of Medicine, Hangzhou, Zhejiang, China; 6 Department of Oncology, Changxing County Hospital of Traditional Chinese Medicine, Huzhou, Zhejiang, China; 7 Department of Oncology, The Second Affiliated Hospital of Zhejiang Chinese Medical University, Xinhua Hospital of Zhejiang Province, Hangzhou, Zhejiang, China

**Keywords:** berberine, colorectal cancer, protocol, randomized controlled trials, recurrence

## Abstract

**Introduction:**

Postoperative recurrence and metastasis of colorectal cancer (CRC) are critical factors affecting long-term survival, and how to prevent postoperative recurrence and metastasis remains an urgent clinical challenge. Studies indicate that berberine has a preventive effect against colorectal adenoma recurrence; however, no research has yet investigated its preventive efficacy against CRC. This trial aims to evaluate the clinical efficacy and safety of BBR in preventing postoperative recurrence and metastasis of CRC by comparing it with a placebo group.

**Methods and analysis:**

This trial is a multicenter, randomized, controlled, double-blind clinical study. A total of 306 eligible patients with stage IIIB, IIIC CRC who had undergone surgery will be randomly assigned in a 1:1 ratio to the placebo group and the trial group. The primary outcome is 2-year disease-free survival (DFS) rate, while secondary outcomes include 1-year and 2-year postoperative recurrence rates of CRC, as well as the incidence of adverse reactions. We expect that this randomized controlled trial (RCT) will provide high-quality evidence on BBR for the prevention of postoperative recurrence of CRC, which can achieve more significant clinical benefits to patients with stage IIIB, IIIC CRC.

**Ethics and dissemination:**

This study has been approved by the institutional review board of The Second Affiliated Hospital of Zhejiang Chinese Medical University (No. 2025-246-IH01).

**Clinical trial registration:**

www.chictr.org.cn, identifier: ChiCTR2600117441.

## 1 Introduction

Colorectal cancer (CRC) represents one of the most prevalent malignant tumors of the digestive tract worldwide and remains a leading cause of cancer-related mortality (1). Recent data from the National Cancer Center (2022) indicate that in China, CRC ranked the second highest incidence among malignant tumors (approximately 10.72%), with a mortality rate ranking fourth (approximately 9.32%), posing a significant public health burden (2). While advances in medical technology have improved early diagnosis and surgical resection rates, postoperative recurrence continues to substantially limit patient survival Clinical evidence shows that approximately 40% of stage III CRC patients experience tumor recurrence and metastasis following radical surgery and standard adjuvant chemotherapy (3). Additionally, the 2-year disease-free survival (DFS) rates demonstrated considerable variation across disease stages: approximately 80% for stage III overall, 79% for stage IIIB, and only 60% for stage III C (4). These findings underscore the urgent need for effect strategies to prevent postoperative CRC recurrence.

Currently, prevention of postoperative recurrence of CRC primarily involves adjuvant antitumor therapy and lifestyle interventions. Evidence suggests that weight management, healthy lifestyle practices, and a diet rich in vegetables, fruits, and whole grains may improve survival outcomes (5). Meanwhile, multiple clinical trials have actively explored therapeutic interventions to reduce recurrence risk-including vitamin D supplementation, postoperative anticoagulation, and nonsteroidal anti-inflammatory drugs (NSAIDs) like aspirin, none have demonstrated their efficacy in large-scale RCTs (6–8). Notably, targeted regulation of gut microbiota has become a research hotspot in the prevention and treatment of CRC progression. Intestinal microbiota dysbiosis appears significantly associated with postoperative recurrence, potentially through mechanisms involving chronic intestinal inflammation secondary to microecological imbalance (9–11).

Traditional Chinese medicine (TCM) has gained increasing recognition in comprehensive cancer care, particularly for its potential to enhancing treatment efficacy while reducing toxicity. Furthermore, guided by the TCM principle of “preventive treatment of disease” and leveraging its multi-target, multi-pathway regulatory strategies, TCM combined with precision medicine may offer novel strategies for postoperative CRC management. Clinical data suggest that long-term TCM administration may reduce CRC recurrence and metastasis risks (12). Specifically, Coptidis Rhizoma, as a commonly used traditional Chinese medicine in clinical practice among CRC patients, especially in those with diarrhea (13). Berberine (BBR) is the main active ingredient of, Coptidis Rhizoma and has shown promising prospects in the treatment of CRC. Clinical trials have shown that BBR can reduce colorectal adenomas recurrence (0.3g twice daily for two consecutive years) with favorable safety profiles (14). Additionally, several studies have confirmed that BBR exerts a significant inhibitory effect on the progression of colorectal adenoma and CRC (15–17). In addition, BBR may be able to overcome cetuximab resistance in CRC, thereby achieving a synergistic effect (18).

Based on the above research findings, our research team has systematically investigated BBR's mechanism in CRC models. Using AOM/DSS-induced inflammation-associated carcinogenesis models, we demonstrated BBR's capacity to inhibit CRC progress by gut microbiota-mediated short-chain fatty acid modulation (19). Building on TCM theory regarding dietary impacts we utilized AOM/DSS to established a CRC model, simulating internal damp-heat syndrome with a high-fat diet. The results identified that BBR significantly upregulated beneficial bacteria, such as Akkermansia and Bifidobacterium and modified lipid metabolism pathways, thereby attenuating high-fat diet-induced CRC progression (16). Additionally, we mechanistically confirmed BBR's protective effects against chemotherapy-induced intestinal mucosal injury via the gut microbiota-mediated PI3K/AKT/mTOR pathway, showing synergistic antitumor effects with 5-Fu (20). However, robust evidence regarding BBR's efficacy in preventing postoperative CRC recurrence remains limited.

## 2 Methods and analysis

### 2.1 Objectives

The primary objective is to evaluate the clinical efficacy and safety of BBR in preventing postoperative recurrence of stage IIIB/IIIC CRC. The secondary objectives are to elucidate the key mechanisms underlying BBR in preventing postoperative recurrence of CRC from the perspectives of gut microbiota and metabolism, screen biomarkers for the early and precise prediction of postoperative recurrence, and provide key targets for the prevention and treatment of CRC progression in the future.

### 2.2 Study design

**Figure 1:**
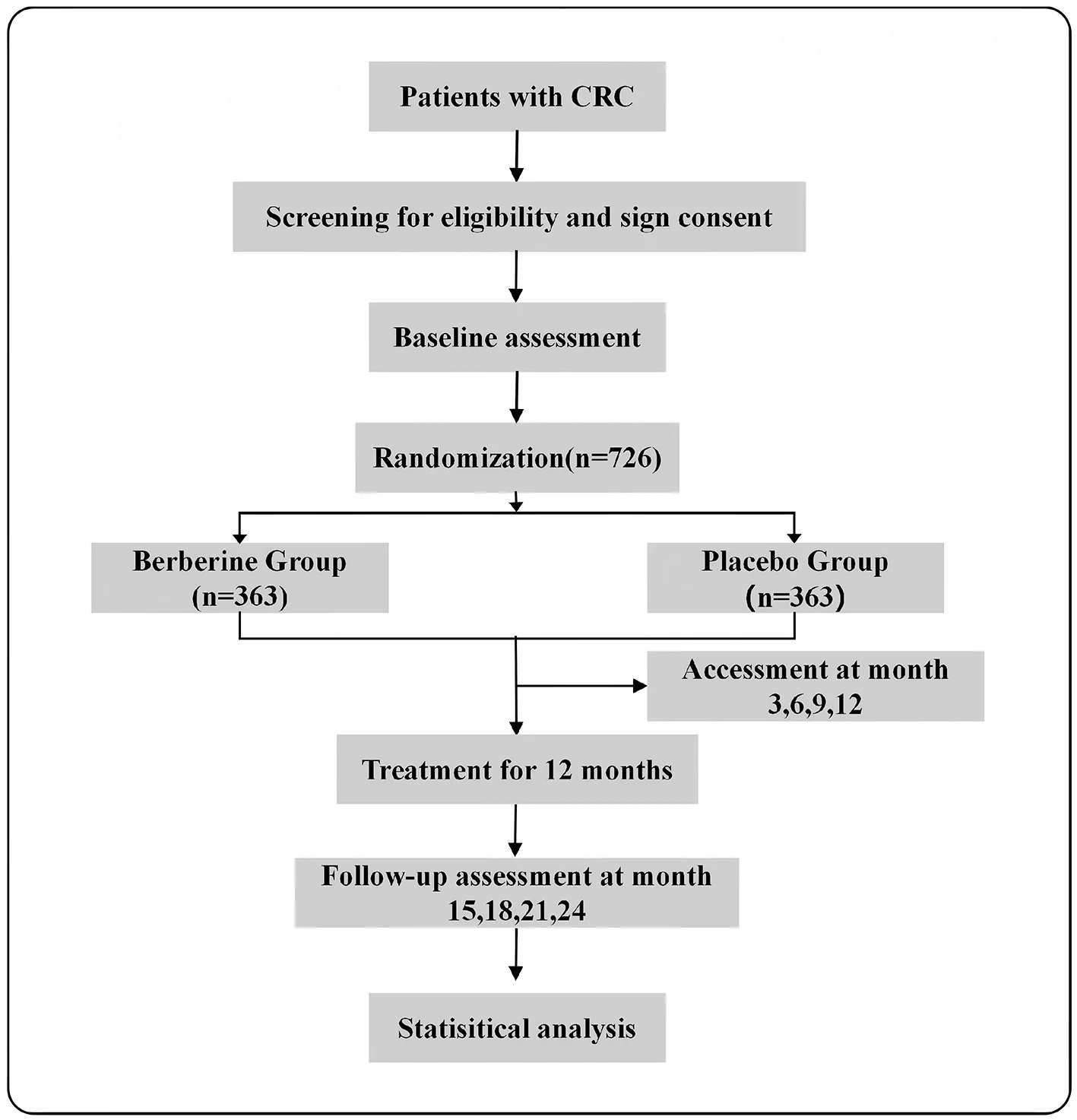
The flow chart of clinical research.

This multicenter clinical trial will be conducted at four tertiary Grade A hospitals within the province. Patients who have undergone surgical resection for stage IIIB/IIIC CRC will be screened and enrolled. Eligible participants will be randomly assigned at a 1:1 ratio to two groups, receiving either oral BBR or placebo at a dosage of three capsules (0.1 g per capsule) twice daily for 12 consecutive months (Figure 1). All patients will receive adjuvant chemotherapy in accordance with standard guideline-based regimens during the study period. Clinical and laboratory data will be collected at baseline, during treatment, and the follow-up phase. This study follows the Declaration of Helsinki and has been approved by the medical ethical committee of the Second Affiliated Hospital of Zhejiang Chinese Medical University (No. 2025-246-IH01). It has been registered on Chinese Clinical Trial Registry: ChiCTR2600117441. This trial protocol was designed and reported in accordance with the SPIRIT 2025 statement. The corresponding checklist is available in the Supplementary material.

### 2.3 Eligibility criteria

#### 2.3.1 Diagnostic criteria

The diagnosis of CRC is based on the Chinese Guidelines for the Diagnosis and Treatment of Colorectal Cancer (2023 Edition) (21), and staging is performed according to the 8th edition of the AJCC TNM staging system.

#### 2.3.2 Inclusion criteria

(1) Patients with pathologically confirmed stage IIIB or IIIC CRC after radical resection, receiving initial treatment;
(2) Aged from 18 to 75 years;
(3) ECOG performance status score less than 2 (22);
(4) Patients who initiate adjuvant chemotherapy within 6 weeks after radical resection of CRC;
(5) No local or distant tumor recurrence was determined by imaging;
(6) Patients with good communication ability who can comply with the study protocol and complete the study procedures;
(7) Patients who have voluntarily signed the informed consent form after fully understanding the study purpose and procedures, disease characteristics, drug effects, related examinations, and potential risks/benefits of this study.

#### 2.3.3 Exclusion criteria

(1) Patients with severe cardiac, hepatic or renal diseases;
(2) Patients with a personal history of other malignancies within 5 years;
(3) Patients with mental disorders, disturbed consciousness or inability to communicate verbally;
(4) Patients with drug allergy or intolerance;
(5) Pregnant women, lactating women or women intending to become pregnant;
(6) Psychiatric patients who are uncooperative.

#### 2.3.4 Termination criteria

(1) Voluntary withdrawal or loss to follow-up by the patient;
(2) Inability to continue participation due to serious adverse events;
(3) Failure to comply with the study plan to complete the scheduled medication treatment and follow-up arrangements:
  1) Investigational drug: For medication adherence assessment in chronic disease studies, the internationally accepted standard is between 80 and 120%. In this study, follow-up visits are conducted every 3 months. Using an illustrative example, the actual number of pills taken (A) is calculated by subtracting the number of pills returned at the visit from the number previously dispensed. The expected pill count is 90 × 2 × 3 = 540pills, yielding an acceptable adherence range of 80% × 540–120% × 540, i.e., rounded to 432–648 pills. If A falls outside this range, the patient is considered non-adherent and will be regarded as a dropout.
  2) Chemotherapy: According to the CSCO guidelines, postoperative adjuvant chemotherapy for stage IIIB, IIIC colorectal cancer should last 4–6 months. Therefore, patients who are intolerant to chemotherapy and receive it for ≤ 3 months will be regarded as dropouts, as this may increase the risk of recurrence and metastasis.

### 2.4 Sample size

This is a RCT with the BBR group as the intervention group and the placebo group as the control group. The 2-year DFS rate in postoperative CRC patients was set as the primary endpoint. According to our previous small-sample observations and literature review, the 2-year DFS rate was estimated to be 67% in the control group and 77% in the intervention group (4). A 1:1 parallel-group design was adopted, with a two-sided type I error α = 0.05 and a test power (1–β) = 0.8. Sample size calculation was performed using PASS 2021 software, yielding a required sample size of 316 patients in the intervention group and 316 patients in the control group. Considering a 15% dropout rate due to loss to follow-up and refusal, at least 363 patients were needed in each group, resulting in a total target sample size of 726 patients.

### 2.5 Randomization, allocation, and blinding

#### 2.5.1 Randomisation and allocation

The randomization sequence is generated by an independent third party to ensure unpredictability. A central randomization system is used to prevent investigators from knowing the group assignments in advance. Before the trial commenced, pre-prepared blinded drugs and corresponding emergency letters were allocated to each center in consecutive number segments. Drug dispensing at each center began with the lowest available number, and drugs were sequentially provided to eligible participants based on their enrollment time.

#### 2.5.2 Blinding

BBR in the intervention group and placebo in the control group are identical in appearance, packaging and labeling (i.e., same color, shape and odor). Participants are not informed of the actual group assignment. Investigators/assessors are not involved in random allocation, and only record data according to the assigned numbers.

The blinding procedure is supervised throughout by a statistical expert. The implementation of blinding is carried out by staff who are not directly involved in the trial's randomization and drug administration processes. First, the personnel performing the blinding verify the consistency of the appearance and packaging of the study drugs across all groups; subsequently, labels bearing the corresponding drug numbers are affixed to the outer packaging of the respective drug groups. The entire blinding process is documented in detail and securely stored.

The emergency unblinding letters, which contain the group assignment information corresponding to the drug codes, are sealed and distributed to each study center together with the pre-prepared blinded drugs. In an emergency situation, if the investigator deems it necessary to identify the administered drug for the management of an adverse event, unblinding may be performed. The investigator performing the unblinding must document in detail the reason, the date, and his/her signature upon opening the emergency unblinding letter.

Data Monitoring Committee (DMC) independently reviews unblinded data to ensure safety. Unblinding is performed jointly by the independent third party and investigators after study completion or in case of an emergency, to clarify the group allocation.

### 2.6 Interventions

Patients will be administered orally at a daily dose of 600 mg (300 mg twice daily, orally), continued for 1 year in the BBR group; and the control group will receive identical-appearing placebo tablets administered at the same dosage regimen (300 mg twice daily) for the same duration. Berberine hydrochloride tablets (0.1 g per tablet) and matching placebo tablets (principal component: starch) were both manufactured and supplied by Chengdu Jinhua Pharmaceutical Co., Ltd.

Conventional Chemotherapy Regimens include the following (23, 24): the single-agent regimen consists of Capecitabine 1,250 mg/m^2^ administered orally twice daily on days 1–14, repeated every 3 weeks; sLV5FU2 involves Leucovorin (LV) 400 mg/m^2^ intravenously over 2 h on day 1, followed by 5-FU 400 mg/m^2^ as an intravenous bolus on day 1, and then 5-FU 1,200 mg/(m^2^·d) via continuous intravenous infusion for 2 days (total 2,400 mg/m^2^ over 46–48 h), repeated every 2 weeks; CAPEOX (also known as XELOX) comprises Oxaliplatin 130 mg/m^2^ intravenously over 2 h on day 1 and Capecitabine 1,000 mg/m^2^ orally twice daily on days 1–14, repeated every 3 weeks; mFOLFOX6 consists of Oxaliplatin 85 mg/m^2^ intravenously over 2 h on day 1, Leucovorin (LV) 400 mg/m^2^ intravenously over 2 h on day 1, 5-FU 400 mg/m^2^ as an intravenous bolus on day 1, and then 5-FU 1,200 mg/(m^2^·d) via continuous intravenous infusion for 2 days (total 2,400 mg/m^2^ over 46–48 h), repeated every 2 weeks; and FOLFOXIRI involves Irinotecan 165 mg/m^2^ intravenously on day 1, Oxaliplatin 85 mg/m^2^ intravenously on day 1, Leucovorin (LV) 400 mg/m^2^ intravenously on day 1, and a total of 2,400–3,200 mg/m^2^ 5-FU via continuous intravenous infusion over 48 h on day 1, repeated every 2 weeks.

### 2.7 Outcome measures and follow-up

#### 2.7.1 Outcome measures

The primary endpoint is 2-year disease-free survival, defined as the proportion of enrolled patients who develop disease recurrence or death within 2 years following randomization. Recurrence is categorized as either local or distant. Local recurrence is defined as the histologically confirmed reappearance of tumor consistent with the primary lesion at the anastomosis, adjacent bowel, surgical incision, mesentery and lymph nodes, abdominopelvic peritoneum, or retroperitoneum. Distant recurrence refers to metastatic spread of the tumor to distant organs including the liver, lungs, brain, and other extrapelvic sites.

#### 2.7.2 Secondary outcome measures

Secondary outcome measures include the 1-year and 2-year recurrence and metastasis rates, as well as the incidence of adverse events. Gut microbiome analysis will be performed using 16S rRNA gene sequencing to compare differences in gut microbial diversity and the abundance of characteristic genera between groups, and metabolomic analysis will be conducted to explore the correlation between serum and fecal metabolic profiles and clinical efficacy.

All assessments will be performed at baseline and every 3 months for 2 years following enrollment, or until disease recurrence or metastasis is observed in participants.

#### 2.7.3 Follow-up program

Following the provision of written informed consent, baseline data and biological specimens will be collected from eligible patients in this study. Specifically, demographic data (sex, age), physical examination findings, vital signs, medical history, comorbidities, Healthy Eating Index-2020 (HEI-2020) and tumor characteristics (tumor type, pathological features, metastatic sites, and TNM stage) will be documented via case report forms (CRFs). In addition, routine blood tests, biochemical measurements, tumor marker assays, and imaging examinations will be performed, and clinical biospecimens including blood and fecal samples will be collected.

**Figure 2:**
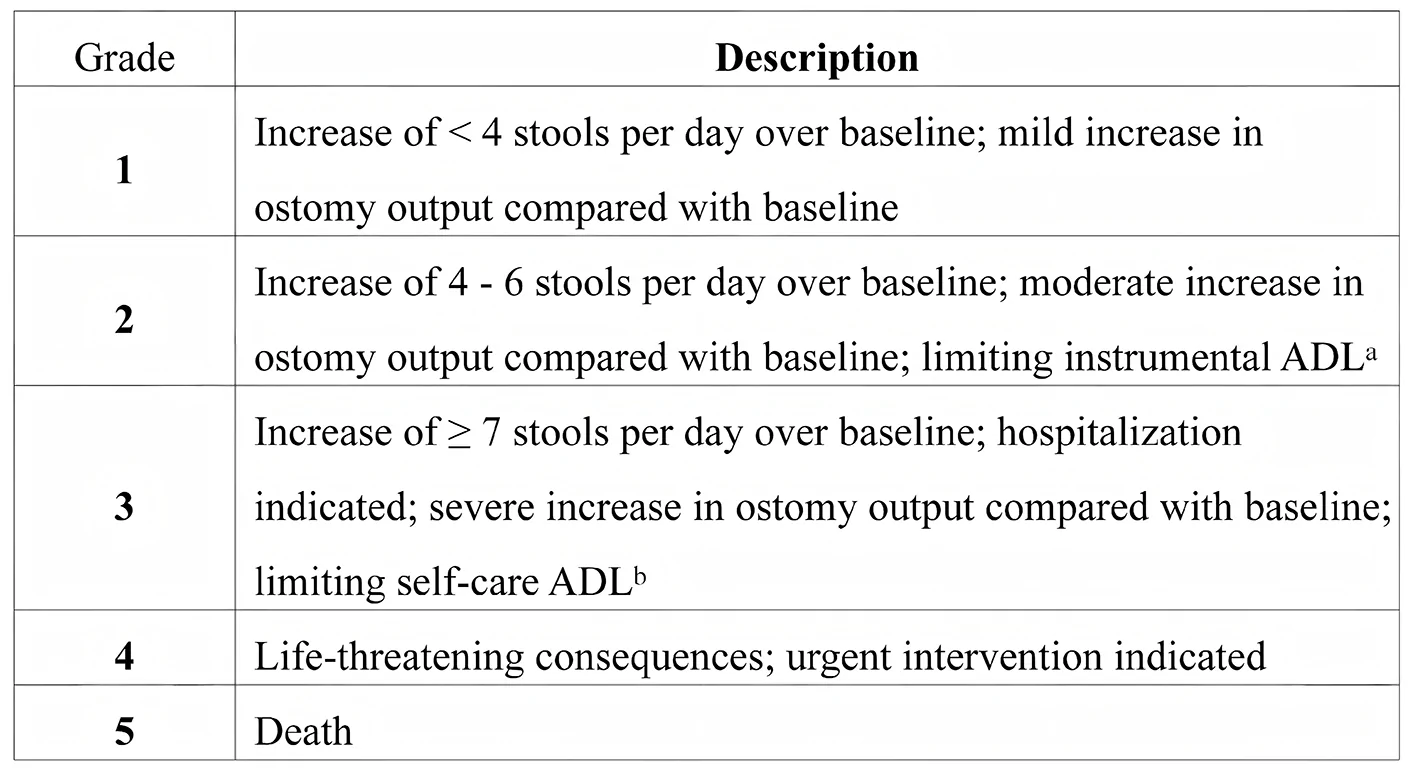
NCI-CTCAE Diarrhea Grading Scale. This table is based on the National Cancer Institute's (https://dctd.cancer.gov/research/ctep-trials/for-sites/adverse-events) Common Terminology Criteria for Adverse Events (NCI-CTCAE v5.0) (36), the standard classification used in oncology . ADL, activities of daily living. Definition: A disorder characterized by an increase in frequency and/or loose or watery bowel movements. ^a^Instrumental ADL refers to preparing meals, shopping for groceries or clothes, using the telephone, managing money, etc. ^b^Self-care ADL refers to bathing, dressing and undressing, feeding self, using the toilet, taking medications, and not bedridden.

During the treatment period, regular clinical assessments will be performed, including evaluation of tumor status, occurrence of adverse events, incidence of grade ≥2 diarrhea (Figure 2), chemotherapy dose reductions or delays, and medication adherence. Routine laboratory tests will also be conducted, including complete blood count, biochemical tests, and routine stool examination, as well as imaging examinations consisting of chest-abdomen-pelvis CT or contrast-enhanced abdomen-pelvis CT/MRI. Additionally, if a participant takes any medication other than the chemotherapy agents and the investigational drug, they must report the name, dosage, and duration of treatment to the physician at the follow-up visit. These data will be recorded in the CRF. At the completion of the 1-year treatment period, all participants will undergo standard colonoscopy, and blood and fecal samples will be collected.

Meanwhile, due to factors such as chemotherapy, subjects may experience grade 2 or higher diarrhea during the study period, so we need to be highly cautious and develop effective treatment plans in advance. When grade 2 or higher diarrhea occurs, emergency medications such as smectite powder, loperamide, or octreotide, should be administered after evaluation by physician. The investigator must record in the CRF detailed information, including the diarrhea grade, name of the rescue medication, dosage, and time of administration. If the patient experiencing Grade 3 diarrhea, the investigational drug will be temporarily discontinued until the toxicity resolves to Grade 1 or baseline, upon which it may be resumed. In addition, once a patient experiencing Grade 4 diarrhea, the investigational drug must be permanently discontinued and the subject discontinue the study.

During the follow-up period, regular assessments of tumor status will be performed, along with routine laboratory examinations including complete blood count, biochemical tests, and routine stool tests, as well as imaging examinations consisting of chest-abdomen-pelvis CT or contrast-enhanced abdomen-pelvis CT/MRI. For each quarterly follow-up visit, a window period of ±7 days is permitted. If a patient is unable to attend the hospital visit, conduct a telephone follow-up and remind the patient to complete the routine check-up. At the end of the 1-year follow-up period, a standard colonoscopy will be performed again, and blood and fecal samples will be collected.

In addition, if disease recurrence or metastasis occurs in the patient during treatment and follow-up, sample collection shall be performed at the time of recurrence and metastasis.

#### 2.7.4 Study schedule

**Figure 3:**
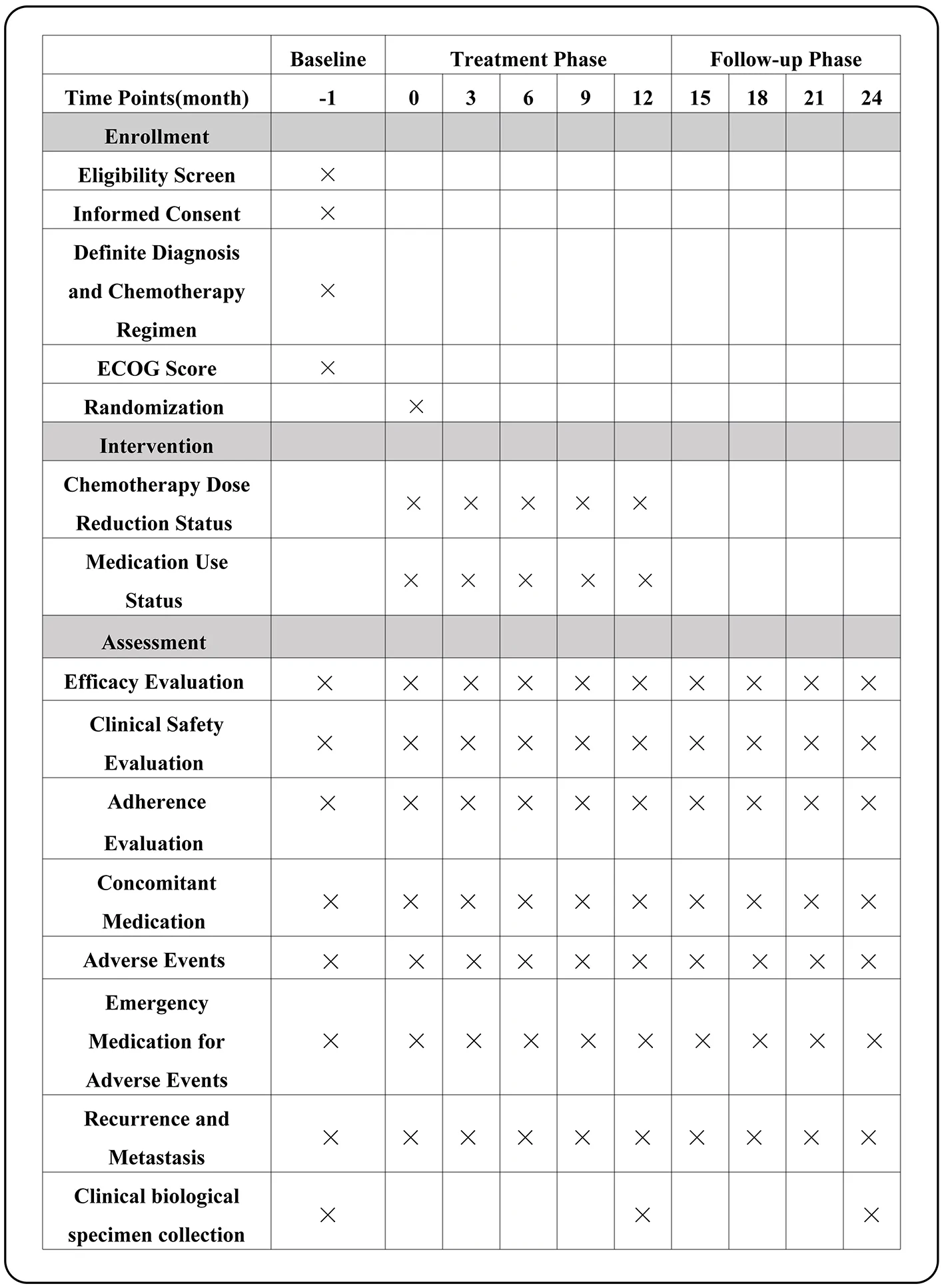
Schedule of enrollment, intervention, and assessments. If the patient experiences recurrence or metastasis within 2 years after enrollment, sample collection should be performed at the time of recurrence or metastasis.

The study treatment and assessment plan are summarized in Figure 3.

### 2.8 Data management and monitoring

We will use the CRF for data collection, which includes general information laboratory examinations, health costs, and so on. Researchers will ensure that the CRFs are filled out accurately and completely. Any incorrect records will be re-filled with the correct information, along with the researcher's signature and date of the day.

A database will be established, encompassing information such as basic patient demographics, diagnosis and treatment details, and imaging assessments. The data of enrolled patients will be reviewed on a regular basis. If any suspicious or discrepant data are identified, the investigator will be contacted for discussion and correction of the data. The database will be locked after the blinded data review report is completed. Quality control-related documents shall be retained, including original records of data consistency checks, range and logic checks, original records generated during the blinded review, and query logs of communications between investigators and monitors.

#### 2.8.1 Data monitoring

Monitors will conduct regular follow-up visits in accordance with quality control requirements. Source data will undergo 100% dual review. Any discrepant data must be submitted in a data correction report and will be corrected after review.

### 2.9 Statistical analysis

First, normality tests were performed on the measurement data. For data conforming to a normal distribution, parametric statistical methods including *t*-test, paired *t*-test, analysis of variance, and analysis of covariance were used. For non-normally distributed data, nonparametric methods such as rank-sum test and paired rank-sum test were adopted. Enumeration data were analyzed using chi-square test and Fisher's exact test, while ordinal data were analyzed using Ridit analysis, CMH test, or other nonparametric methods. In the multicenter comprehensive efficacy analysis, the CMH test was used for enumeration data, and analysis of covariance was used for measurement data. All statistical analyses were performed using SAS 9.3 software. For patients with missing data due to dropout or loss to follow-up, the last observation carried forward method may be used for imputation, followed by a sensitivity analysis of the results. All statistical tests were two-sided, and P < 0.05 was considered statistically significant.

### 2.10 Adverse events

All adverse events (including side effects such as gastrointestinal reactions and allergic reactions) will be documented in detail and graded throughout the study period. When a serious adverse event occurs, the investigators will provide all necessary treatment measures and report the adverse event to the Ethics Committee of the Second Affiliated Hospital of Zhejiang Chinese Medical University.

## 3 Discussion

Postoperative recurrence and distant metastasis remain a formidable clinical challenge in the management of CRC, particularly for patients with advanced-stage disease, in whom recurrence rates are markedly elevated and long-term prognosis is severely compromised. A previous RCT with long-term follow-up demonstrated that BBR may exert a potential preventive effect on colorectal adenoma postoperative recurrence, with its protective effect persisting for up to 6 years or even longer (25). Another study has shown that BBR can exert preventive and therapeutic effects on CRC by binding to SIGMAR1 (26). Building on these prior clinical findings, the present study specifically focuses on patients with stage IIIB and IIIC CRC. Through standardized intervention and long-term follow-up, we aim to further evaluate the preventive efficacy and potential of BBR against postoperative recurrence in this high-risk advanced CRC patient population.

Blood and fecal samples will be collected at baseline, 1-year treatment, 1-year follow-up or upon the occurrence of recurrence and metastasis for multi-omics analyses, specifically metagenomics (16S rRNA sequencing and shotgun metagenomics) and metabolomics (untargeted and targeted approaches). These samples will be used for comprehensive metabolic and mechanistic studies centered on gut microbiota-metabolite interactions and their roles in CRC recurrence.

In recent years, accumulating evidence has underscored the pivotal role of gut microbiota dysbiosis in CRC initiation, progression, and recurrence (27). The gut microbiome and its metabolic products are now recognized as critical regulators of tumorigenesis, immune evasion, and therapeutic response in CRC patients. Specifically, CRC recurrence has been associated with distinct microbial signatures. On the one hand, enrichment of pathogenic oral pathobionts has been observed in CRC patients-Parvimonas micra, Fusobacterium nucleatum, and Peptostreptococcus stomatis were found to be significantly more abundant in tumors with microsatellite instability, forming a distinct bacterial network linked to colorectal carcinogenesis (28). On the other hand, depletion of beneficial commensals is also a hallmark of CRC-associated dysbiosis. Faecalibacterium prausnitzii and Akkermansia muciniphila are recognized as next-generation probiotics with anti-inflammatory and barrier-protective properties, and their reduced abundance has been documented in CRC patients (29). These microbial alterations contribute to a pro-tumorigenic microenvironment by promoting chronic inflammation, producing genotoxins (e.g., colibactin), and disrupting bile acid metabolism (26). Complementing gut microbiome research, metabolomics has emerged as a powerful tool for investigating CRC recurrence mechanisms and identifying non-invasive biomarkers. Gut microbial metabolites, including short-chain fatty acids (SCFAs), bile acids, tryptophan derivatives, and polyamines, play role in CRC recurrence: they can serve as early warning indicators for disease progression (30).

The potential molecular mechanism by which BBR prevents postoperative recurrence of CRC can be comprehensively explored through integrated gut microbiota and metabolomics analyses. Previous studies have confirmed that BBR exerts its anti-tumor effects by: (1) reshaping gut microbiota composition, which is manifested by increasing the Bacteroidetes/Firmicutes ratio in most CRC models and clinical studies, promoting the enrichment of SCFA-producing bacteria (e.g., Faecalibacterium and Roseburia), and inhibiting the proliferation of pathogenic species such as Fusobacterium nucleatum (31, 32); (2) modulating systemic bile acid homeostasis in a dose-dependent manner, and suppressing the accumulation of tumor-promoting secondary bile acids, including deoxycholate (33); (3) triggering GPX4-dependent ferroptosis and impairing mitochondrial energy metabolism (34); (4) regulating key signaling pathways involved in tumor progression (e.g., Wnt/β-catenin and NF-κB) (16, 35). These findings provide an important foundation for our integrated metagenomics and metabolomics approach to unravel the core mechanisms underlying BBR's anti-recurrence effects.

This study is a multicenter, double-blind RCT with high-level evidence and strong reliability. It aims to explore a feasible therapeutic regimen for the prevention of postoperative recurrence of CRC.

Despite the potential geographical limitation of our enrolled participants, who are predominantly from the eastern coastal regions of China, this multicenter, double-blind RCT still provides high-level evidence to support the preventive efficacy of BBR against CRC postoperative recurrence. More importantly, our study is designed to elucidate the core mechanistic pathways by which BBR exerts its anti-recurrence effects, including the modulation of gut microbiota-metabolite crosstalk and the regulation of key signaling pathways involved in tumor metastasis. These findings will not only enrich the theoretical basis for BBR application in CRC clinical management but also provide novel strategies and actionable targets for the development of targeted preventive interventions for advanced CRC patients. Collectively, this study holds significant clinical translational value, aiming to optimize the postoperative recurrence prevention strategy for CRC and improve the long-term survival and quality of life of patients.

### 3.1 Trial status

The study is scheduled to enroll patients starting from February 1, 2026, and is expected to conclude on December 31, 2028. As of the time of writing, subject recruitment is gradually underway. The four research centers will collaborate on subject recruitment, intervention implementation, follow-up evaluation, and data collection.
